# Supplementary material for: Ethical care in patients with Covid-19: A grounded theory
Source: PLoS One. 2024 Mar 28;19(3):e0300156. doi: 10.1371/journal.pone.0300156 (PMC10977892; doi:10.1371/journal.pone.0300156)
Supplement: S1 Table — (DOCX) [file pone.0300156.s001.docx]

| **S1 Table: The first version of the interview guide** |
| --- |
| - Please describe one day of your experience in caring for a patient with Covid-19? |
| - What is the difference between caring for patients with Covid-19 and other patients? |
| - What aspects of care do you pay attention to when caring for a patient with Covid-19? |
| - What does ethical care mean to you? |
| - What conditions exist in your department that make it possible to provide ethical care? |
| - What conditions exist in your department that prevent you from providing ethical care? |
| - Has there ever been a situation where your decision to care went against the patient's or your colleague's opinion or hospital policy? |
