# Supplementary material for: Ethical care in patients with Covid-19: A grounded theory
Source: PLoS One. 2024 Mar 28;19(3):e0300156. doi: 10.1371/journal.pone.0300156 (PMC10977892; doi:10.1371/journal.pone.0300156)
Supplement: S1 File — (DOCX) [file pone.0300156.s002.docx]

In the name of god

| **S1 File. Primary codes** |
| --- |
| **1- Eliminate the patient's fear and anxiety** |
| Reducing stress by saying soothing words to the patient |
| Reducing the patient's stress with the help of psychological counseling |
| Reducing stress by hiding the reason for hospitalization from the patient |
| Reduce stress by talking to the patient |
| Reducing stress by explaining the recovery process and the course of the disease to the patient |
| Reducing the patient's stress with soothing talks |
| Reducing stress and anxiety by talking to the patient |
| Reducing patient stress by being available to the patient |
| Reducing the patient's stress by cohabiting the better patient with the worse patient |
| Reducing stress by allowing a companion to stay with the patient |
| Reducing stress by supporting the patient |
| Reducing anxiety and fear by explaining the treatment process to the patient |
| Reducing the patient's anxiety by helping him |
| Reducing patient anxiety with education |
| Reducing the patient's anxiety and worry by diverting thoughts |
| Reducing the patient's anxiety through the presence of a companion |
| Reducing patient anxiety and stress |
| Reducing fear by sharing experiences of getting worse patients |
| Reducing fear in the patient by joking |
| Trying to reduce the patient's anxiety by making the disease look easy |
| Help reduce stress by feeding the patient |
| Try not to stress the patient |
| Providing the necessary conditions to reduce fear in the use of bi-pep |
| Try not to add stress and pain to the patient |
| Calming and psychologically supporting the patient by touching his hand |
| Creating mental peace and reducing the fear of death by educating the patient |
| Creating relaxation by placing sober people in the same room to calm each other down |
| Calming the patient by talking to him |
| Calming down a stressed patient by asking for a sedative |
| Relaxation by giving sedatives |
| Create relaxation by allowing the patient to play music |
| Calming the patient by talking to the nurse |
| Calming the patient by comforting and explaining to the patient |
| Calming the patient by giving sedative pills |
| Calming and convincing the patient by joking |
| Bringing relaxation to the rooms with more defiance |
| Creating comfort by placing the patient's bed near the door of the room |
| Calming the patient by talking |
| Calming companions by talking to them to better manage the situation |
| Calming the patient by talking |
| Calming the patient as much as possible by talking to him |
| Creating relaxation at the request of the psychologist in times of severe restlessness of the patient |
| Calm the patient by encouraging them to be calm |
| Relaxing the stressed patient by spending more time with him |
| Creating peace in the patient by playing the role of a psychologist |
| Creating peace in the patient by trivializing the disease |
| Calming the patient by saying that you will get better |
| Calming the patient by answering his questions |
| Calming the patient by explaining the environment to the patient |
| Calming the patient with the help of a companion |
| Creating relaxation by inviting the patient to relax |
| Creating relaxation by inviting the patient to relax |
| Creating relaxation by inviting the patient to relax |
| Creating peace by expressing the same way of caring for the patient and the family |
| Creating peace by having the right behavior with the patient |
| Calming the patient with the presence of his companion |
| Creating comfort for the patient by meeting his needs |
| Creating peace by allowing family visits |
| Calming patients |
| Calming the patient by joking |
| Try to calm the patient |
| Try to calm the patient |
| Try to calm the patient |
| Giving sedatives to stressed patients to sleep well at night |
| Giving mental and emotional peace to the patient |
| Reduce anxiety by talking to the patient |
| Reduce patient anxiety by stating similar improvements |
| Reducing the patient's anxiety about being alone by instilling confidence in him |
| Reducing patient anxiety by instilling continuous patient care |
| Reduce the anxiety of the anxious patient by showing the patient being discharged |
| Reducing anxiety by encouraging the patient to get better |
| Reducing patient anxiety by showing objective documentation |
| Reducing restlessness by changing the patient's bed to talk to his companion through the window |
| Relieving the patient's mind by talking to the patient |
| Ease the patient's mind by getting help from the family |
| To ease the patient's mind in terms of better treatment in ICU |
| Reassuring the patient by explaining about drug |
| Creating a sense of comfort in the patient |
| Psychological support by communicating with the patient |
| Request for psychological counseling for patients who were hospitalized for more than 5 days |
| Psychiatric consultation |
| Using psychotherapy for the patient |
| Making the patient's heart stronger by leaving the light on with low light at night |
| Provide encouragement by opening the window of the patient's room to see the family |
|  |
| **2- Creating comfort and physical comfort for the patient** |
| Considering the comfort of the patient regarding the location of vein extraction |
| Creating comfortable conditions by changing the patient's position |
| Giving cotton and comfortable clothes to the patient |
| Asking companions to provide comfortable underwear on a daily basis |
| Making the patient more comfortable by implementing the hourly hospitalization order of patients |
| Keeping in mind the comfort and benefit of the patient to put in bi-pep |
| Creating comfort by changing the patient's resting place according to his request |
| Changing the problematic bed of the patient to create more comfort |
| Creating more comfort by quickly meeting the patient's needs |
| Asking the patient's opinion about the location of the venipuncture, which is easier |
| Allowing the patient to adjust the position for the IV |
| Allowing attendants to bring what the patient craves |
| Allowing companions to bring flasks of tea |
| Allowing companions to bring food from home |
| Allowing the clergy to do the basic work of the patients |
| Provision of care and personal items for the patient's comfort by the family |
| Respecting the personal habits of the patient to ensure his well-being |
| Taking measures for the comfort of the patient |
| Helping to make the patient's necessary calls from the ward's phone |
| Transmitting the words of the patient to the family through the phone |
| Meeting the needs of patients with the help of companions |
| Meeting the patient's needs |
| Meeting the patient's request |
| Relieving the patient's need for repeated painkiller injections |
| Providing a phone to make a video call with the family |
| Relieving the patient's boredom by making a video call |
| Dialing a number for the patient to talk to the family |
| Bringing a wheelchair for the patient to go to the bathroom and perform ablution |
| Giving a rug to the patient to pray |
| Giving mohr to the patient to pray |
| Giving my team's seal and stone to pray |
| Giving religious books to the patient |
| Giving seal to the patient for prayer |
| Giving my team stone to the patient |
| Giving the rug to the patient |
| Giving life to the patient to start the patient's work |
| Showing the location of the seal and the prayer hall for praying |
| Show the qibla section |
| Asking the companion to bring religious books for the patient |
| Waking up the patient for morning prayer if entrusted to the nurse |
| Being a facilitator for performing the patient's religious duties |
| Bringing tea for the patient from the hospital's alehouse |
| Feeding a patient with low saturation |
| Feeding the patient despite not having the duty to feed |
| Feeding an unaccompanied patient |
| Asking other attendants about giving water to an unaccompanied patient |
| Bringing water to the patient even in crowded times |
| Giving water and food to the patient who is not accompanied |
| Bringing water to the patient |
| Bringing water and tea to the patient |
| Prepare a spoon for the patient |
| Provide patient requests |
| Fulfilling the patient's demand such as water |
| Fulfilling the patient's wishes as much as possible |
| Not being indifferent to the patient's request |
|  |
| **3- Creating life expectancy in the patient** |
| Giving hope to the patient by comparing the patient's condition with previous days |
| Giving hope to the patient by hiding the sick patient from other patients |
| Giving hope to the anxious patient |
| Giving hope to the patient by making recovery conditional on factors |
| Giving hope to patients at the end of a difficult time |
| Giving hope to the patient with reason |
| Giving hope to the patient with positive and hopeful sentences |
| Giving hope to the patient by showing sick patients who got better |
| Giving concrete examples of getting better |
| Giving hope to the patient by saying that you will get better |
| Hoping the patient to recover |
| Giving hope to the patient by teaching and explaining previous experiences about other diseases |
| Give hope to the patient |
| Giving hope to the patient by exaggerating small improvements |
| Giving hope to the patient by implementing new procedures |
| Giving hope to the patient with daily positive changes |
| Hoping that the patient has a chance to recover |
| Giving hope to the patient by encouraging him positively |
| Hope the patient to speak in a relaxed state |
| Giving hope to the patient with encouraging words |
| Emphasis on the improving process |
| Giving hope to the patient to improve his mental state |
| Giving the patient hope for recovery |
| Hope for a sick patient |
| Giving hope to the patient despite not being sure of the outcome of the treatment |
| Giving hope to the patient despite the bad prognosis of the disease |
|  |
| **4- Improving the patient's vitality** |
| Improving the condition of the patient by allowing the family to visit the patient |
| Improvement of the patient's condition by allowing a short face-to-face meeting with the companions |
| Enhance the patient's energy by talking to the patient |
| Enhancing the patient's energy by connecting the patient with the family |
| Improving the morale of the patient by allowing the companion to be with the patient |
| Improving patient morale by using therapeutic communication techniques |
| Changing the patient's mood by having an appointment |
| Maintaining morale and helping the patient by meeting first-class family members |
| Maintaining the patient's morale by hiding bad news from the patient |
| Improving the condition of the patient by allowing the family to visit the patient |
| Improvement of the patient's condition by allowing a short face-to-face meeting with the companions |
| Enhance the patient's energy by talking to the patient |
| Enhancing the patient's energy by connecting the patient with the family |
|  |
| **5- Spiritual help to the patient** |
| Encouragement to send blessings |
| Encouragement to read Ayatul Kursi |
| Encouragement to appeal to imams |
| Teaching the patient to pray |
| Expressing the kindness of God to reduce the fear of sins |
| Allowing jihadists to provide spiritual support to patients |
|  |
| **6- Patient and family awareness** |
| Educational advice to patients |
| Educational advice about effective cough |
| Educational recommendation about how to do deep breathing |
| Educational recommendation based on discovered needs |
| Educational advice about getting in the right position to breathe better |
| Educational advice on care tips for diabetic patients |
| Educational advice about diet to the patient |
| Educational advice to the patient during transfer to another ward |
| Educational advice to the patient when the number of patients is low |
| Educational advice about diet to the patient in reducing the side effects of drugs |
| Educational advice as much as possible due to changes in wards |
| Educational advice about foods that increase sugar |
| Educational advice to the patient about the type of position during sleep |
| Educational advice about being in a semi-sitting position |
| Educational advice and patient follow-up during discharge |
| Educational advice to the patient regarding the use of nurse call |
| Educational advice to the patient about the complications of blood transfusion |
| Educational advice of nurses call to patients |
| Educational advice about diet |
| Educational advice about diet |
| Educational advice to companions to speak well with the patient |
| Educational advice focusing on patient activity |
| Educational recommendation to walk around the bed to improve breathing |
| Educational advice for breathing exercises |
| Educational advice for removing nail polish |
| Educational advice for walking around the bed |
| Educational recommendation for masking the patient |
| Educational recommendation for masking the patient |
| Educational recommendation to consume fruit to juice due to less sugar |
| Educational advice for treating patients at home due to drug side effects |
| Educational advice on how to sleep due to unilateral lung involvement |
| Educational recommendation with the patient to place the water bottle next to the patient |
| Educational advice to change the position from lying to sitting |
| Educational advice about the changes in tests to the patient |
| Give adequate educational advice |
| Helping the patient to buy the necessary medical equipment |
| Helping the patient to ask his questions to the doctor |
| Helping the patient to give or not to give personal consent |
| Guidance to the patient in making the right decision |
| Guidance with the patient about how to transfer a sick patient |
| Helping companions to inquire about the patient's condition through other relatives |
| Guidance for changing the patient's doctor in case of patient dissatisfaction |
| Patient guidance to find answers to questions |
| Guidance with the patient about the patient's discharge |
| Providing information about the patient's discharge time |
| Providing information about the possibility of referring to the vice president of treatment for complaints |
| Providing information on the course of the disease along |
| Providing information to patients about their treatment process |
| Providing information to the companions of forgetful patients |
| Providing information about your working hours to the patient |
| Providing information about any procedure being performed for the patient |
| Providing information to patients about medications |
| Providing information about the hospital environment |
| Providing information about the units of the ward when the patient enters the ward |
| Providing information about new drugs and their side effects to the patient |
| Providing information in writing to the patient who forgets to work |
| Providing information about the type of injectable drug to the patient |
| Providing information to patients about their illness |
| Providing information to cancer patients with caution and their own will |
| Providing information about the benefits of medicines |
| Providing information about the patient's religious questions |
| Providing information about the type of injectable drug |
| Providing information about the type of injectable drug by providing objective documentation |
| Provide information about the drug before asking the patient |
| Providing information about the type of injectable drug to the patient |
| Providing information about injectable drugs |
| Provide information to the patient about compression before the procedure |
| Providing information to clear misunderstandings from the patient's companion |
| Providing information about the condition with the patient after calming down |
|  |
| **7-Convince the patient** |
| Convincing the patient by explaining the reason for doing things |
| Convincing the patient by explaining the logical reason for the need to have a catheter |
| Convincing the patient about the reason for using bi-pep for him |
| Convincing the patient to use oxygen using objective documentation |
| Convincing the patient by answering his question |
| Convincing the patient to be sent to another hospital by talking to him |
| Convincing about the prevention of complications of going to the bathroom without oxygen |
| Convincing the patient with explanations and reasons |
| Convincing the patient by explaining the drugs to him |
| Trying to convince the patient to cooperate |
| Try to convince the patient who does not consent to the procedure |
| Try to convince the patient |
| Trying to convince the patient by explaining the nurse's actions |
| Trying to convince the patient by showing objective documentation |
| Try to convince the patient about bed rest |
| Trying to convince the patient who did not want to swear |
| Trying to convince the patient about the side effects of not giving a blood transfusion |
| Trying to convince the patient to do endoscopy |
| Trying to convince about the risks of transfer or early discharge of the patient |
| Trying to accept the patient by asking the doctor for help |
| Trying to convince the companion not to smoke and not to complain about the patient |
| Trying to convince the patient to follow the treatment regimen |
| Trying to accept the patient to comply with the treatment process |
| Trying to satisfy the patient who did not allow blood collection by bringing an expert |
| Trying to satisfy the patient by explaining the reason for not having a nurse of the same gender as the patient |
| Trying to satisfy the patient by explaining the reason for frequent blood draws |
| Trying to satisfy the patient by explaining the reason for not removing the vein from the hand |
| Try to satisfy the patient by explaining why it is not appropriate to take a vein from the hand |
| Try to satisfy the patient by explaining the need to use nasal spray instead of nasal spray |
| Trying to satisfy the patient by explaining the reason for frequent blood draws |
| Try to satisfy by explaining the need for good nutrition and improving the patient's condition |
| Try to satisfy by explaining the possible risk of misplaced transfer of the patient |
| Trying to satisfy by explaining the reason for not injecting too much painkiller |
| Try to satisfy the patient by explaining |
| Talking logically with the patient and convincing him |
| Convince the patient to do the procedure by giving him a warning |
| Satisfying the patient by talking to him |
| Try to resolve misunderstandings about the patient's treatment process |
| Trying to resolve the patient's misunderstanding by explaining the reason for the patient's late arrival |
|  |
| **8- Helping patient’s companions** |
| Allowing two companions to stay for one patient |
| Not charging co-payments from patients |
| Taking extra food from the kitchen for companions |
| Giving the extra food untouched by the nurses along with the patient |
| Giving bed to companions |
| Giving a personal phone number to the patient's companions |
| Increasing interaction with the patient |
| Delivery of food brought by companions to patients |
| Providing a resting place for companions |
| Paying attention to the patient's companion |
| Reconciling with the patient's companion and getting him out of his heart |
| Removing the ignorance of the patient's companions by explaining the work done |
| Relieving the pain of the companions of the deceased patient by handing over the patient's manuscripts |
|  |
| **9- Efforts to gain the patient's trust** |
| Gaining the patient's trust by explaining the side effects of the drugs to the patient |
| Gaining the patient's trust by asking about his physical needs |
| Gaining the patient's trust by seeing the nurse's ability |
| Gaining the patient's trust by telling him the truth |
| Gaining the patient's trust before losing trust |
| Gaining the patient's trust by explaining the reason for the actions to him |
| Gaining the patient's trust by explaining about the drugs |
| Gaining the patient's trust by explaining to him |
| Gaining the patient's trust by paying attention to his needs |
| Gaining the patient's trust by meeting his needs in a timely manner |
| Gaining the patient's trust by communicating properly with him |
| Gaining trust in the patient by observing the experience and dedication of the nurse |
| Gaining the patient's trust by informing the patient about the department and its routine |
| Gaining the patient's trust by seeing the nurse working for him |
| Gaining the patient's trust by understanding his condition |
| Gaining the patient's trust by doing team work |
| Gaining the patient's trust by persuasion by seeing objective documents |
| Gaining the patient's trust with logical answers to the patient's questions |
| Gaining the patient's trust by explaining the procedures to him |
| Gaining the patient's trust by communicating with the patient's companions |
| Gaining the patient's trust by talking to him kindly |
| Gaining the patient's trust by stating the type of injectable drug for him |
| Gaining the patient's trust due to the long presence of the nurse by his side |
| Gaining the patient's trust by seeing the positive performance of the nurse |
| Gaining the patient's trust by talking to him |
| Efforts to win the patient's trust |
|  |
| **10- Obtaining the patient's cooperation to perform care** |
| Attracting the cooperation of the patient by communicating with him |
| Getting the cooperation of the patient by communicating with him |
| Gaining patient cooperation by communicating |
| Attracting the patient's cooperation by answering the patient's questions |
| Attracting the cooperation of the patient with the help of the family |
| Encouraging patient cooperation by joking |
| Encouraging patient cooperation with jokes |
| Encouraging the cooperation of a patient who does not allow blood sampling with a joke |
| Attracting cooperation in the vein of joking |
| Getting the cooperation of the patient by joking to take a blood sample |
|  |
| **11-Financial assistance to the patient** |
| Financial assistance to the patient in treatment costs |
| Forming a group to financially support poor patients |
| Collecting money from ward nurses to treat a poor patient |
| Getting help from a medical assistant to help the patient financially for the invasive procedure |
| Buying equipment for patients by nurses |
| Getting help from charities to cover the expenses of the patient |
| Collecting money among nurses to help the patient financially |
| Referral of the patient to the department of helping patients in need of money in the hospital |
| Providing the possibility of paying the price of drugs in installments for the patient |
| Financial assistance to the patient at personal expense by the nurse |
|  |
| **12- Adherence to professional obligations** |
| Monitor patient with reduced level of consciousness |
| Providing facilities for patients who need special care |
| Spraying the patient by the nurse |
| Drinking water after spraying to prevent mouth infection |
| Not neglecting the care of patients |
| Not neglecting patient care despite the fear of Covid-19 disease |
| Follow up on medication errors to prevent problems |
| Follow-up of patient's affairs from home |
| Follow-up of suction of lung secretions of the patient who was not suctioned |
| Providing urgent and early care to the Covid-19 patient |
| Performing chest physiotherapy of the patient |
| Performing lung physiotherapy for the patient |
| Performing breathing exercises and its effect on the patient's saturation |
| Giving breathing exercises to the patient |
| Performing physiotherapy of the patient's lung |
| Giving a hip to a patient who could not tolerate a Foley catheter |
| Changing dirty or wet bedding without the patient's request |
| Doing the doctor's orders correctly and on time |
| Giving methadone syrup to the patient under the doctor's supervision |
| Responsiveness of the nurse |
| To be answerable |
| Provide care with adequate knowledge and information |
| Providing meticulous care |
| Placing specific patients in direct view of the nurse |
| Providing care according to principles |
| Perform non-harmful actions to the patient |
| Emphasis on not harming the patient |
| Not using gloves to avoid harming the patient |
| Preventing harm to the patient by doing the work properly |
| Bringing a translator to better understand the patient's language and dialect to better provide the caregiver |
| Unification of doctors' orders to prevent drug side effects in patients with Covid-19 |
| Be careful in giving medicine to the patient |
| Be careful in executing orders |
| Be careful in taking the right amount of medicine |
| Doing work for the patient as much as possible |
| Doing all the necessary things for the patient |
| Doing anything for the patient |
| Trying to save the patient's life until the last moment |
| Nurses' efforts to visit the patient on time |
| Asking questions about the patient's problems |
| Obtaining a signature from the patient's companion regarding the condition of the patient's bed sore |
| Use of checklist in patient examination |
| Changing contaminated gloves |
| Replacement of peripheral venous catheter glue due to the possibility of infection |
| Notifying the resident of the error that occurred |
| Notifying the resident of the patient's reduced level of consciousness |
| Inform the doctor to intubate the patient with very low saturation |
| Taking tests from the patient based on logical and scientific principles |
| Acting only in the field of duties |
| Check related tests when medication errors occur |
| Setting up a self-care plan for each patient |
| Doing things for the patient as soon as possible |
| Having a commitment to work in difficult corona conditions |
| Feeling responsible for patients and colleagues |
| Responsibility in doing the right thing |
| Nutritional support of the patient |
| Inquiring about the digestive status of the patient |
| Saturation control at the beginning of the shift |
| Oxygen level control in the first contact with the patient |
| Controlling the patient's saturation level |
| Controlling and recording saturation exclusively during shift delivery |
| Patient oxygen therapy |
| Continuous oxygen therapy of the patient |
| Talking to the patient to distract him from the way he breathes |
| Recording of non-nasal oxygen after the patient walks |
| Warning to the restless patient to put on the mask |
| Asking the patient how to breathe |
| Focusing on the patient's blood oxygen level |
| Focus on oxygen levels |
| Focusing on breathing in caregiving |
| Checking the type of oxygen therapy device |
| Checking the level of respiratory distress in the first contact with the patient |
| Checking the degree of involvement of the patient's lung |
| Giving oxygen continuously |
| Follow up of patients in terms of drug side effects |
| Putting a spoonful of syrup in the patient's mouth |
| The nurse tries to feed the patient |
| Emphasis on feeding on time |
| Special emphasis on nutrition and fluid intake |
| Advice for feeding the patient |
| Use of boiled foods |
| Use of fruit |
| Use of food supplements |
| Use caring touch |
| Coordinating nutritionist for patients |
| A visit by a nutritionist to talk to the patient |
| Sterile injection |
| Correct and sterile drug injection |
| Injection of drugs with infusion sets with burettes and separately |
| Injection of medicine with infusion sets with burettes |
| Inject the drug at the right time |
| Injecting the patient's medicine with the appropriate serum |
| Adherence to Remdesivir injection protocol in febrile patients |
| Checking of medicines by supervisor |
| More monitoring of drug injection in the morning shift due to more numbers |
| Remdesivir injection when more nurses are in the ward |
| Watch out for drug side effects |
| Checking the blood sugar of a patient who is taking corticosteroids |
| Waiting to make sure the patient takes the pills |
| Waiting for the patient to take the medicine |
| Timely administration of Remdesivir |
| Being sensitive to the timing of medication |
| Giving medicines on time |
| Giving medicine without missing a single case |
| Giving medication in the correct and timely manner |
| Giving the medicine to the patient in the correct way |
| Trying to control the side effects of corona drugs |
| Observing safety principles when injecting medicine |
| Paying attention to the accuracy of the dose in the injection of medicine |
| Compliance with the law |
| Compliance with the law |
| Compliance with the law of not allowing meetings |
| Compliance with laws and regulations |
| Compliance with the global protocol for injecting the corona drug |
| Follow the doctor's orders |
| Follow the doctor's orders |
| Compliance with the law prohibiting visits in the ICU ward |
| Fast implementation of the drug |
| Administration of serum and medicine according to the doctor's order |
| Implementation of World Health Organization protocol |
| Dressing the patient based on principles as much as possible |
| Performing CPR as much as possible in a corona patient with severe pulmonary involvement |
| Immediate treatment of the patient's emergency needs |
| Provide care as ordered |
| Observing the rule of hand washing as much as possible due to the existing conditions |
| Notifying the nurse of frequent errors to the supervisor |
| Not allowing the patient to walk around the hospital premises |
| Not allowing to see the family |
| Injection of medicine according to the doctor's order |
| Injection of sensitive drugs only by doctor's order |
| Corona drug injection according to the doctor's order |
| Remdesivir injection according to the doctor's order |
| Failure to register the doctor in the nursing report to disclaim responsibility |
| Written record of the patient's lack of consent who did not allow blood collection |
| A written record of the patient's dissatisfaction that is not justified by the words of the nurse |
|  |
| **13-Truthfulness** |
| Truthfulness in stating the cause of death of other patients |
| Honesty in expressing the effect of Remdesivir |
| Honesty in expressing the lack of effectiveness of drugs to companions |
| Truthfulness in telling the truth to patients |
| Honesty in telling the patient that the prognosis of the disease is not clear |
| Honesty in telling the truth to the patient and family |
| Honesty in stating the reason for not meeting the family |
| Honesty in telling the truth of the prognosis of the disease with the patient |
| Honesty in stating the conditions of effect or lack of effect of drugs on the patient |
| Honesty in the patient's blood oxygen chart and record |
|  |
| **14- Respect patient privacy** |
| Trying to admit female patients in one room |
| Hanging a tent or bedclothes for cauterization |
| Requesting other beds not to look at the catheterization process |
| Placing a shield to cover the revitalization process |
| Protecting the patient's privacy by standing up or changing the patient's position |
| Respect patient privacy as much as possible |
| Respecting the patient's privacy as much as possible |
| Respecting the patient's privacy |
| Preservation of patient privacy |
| Protecting the privacy of the agitated patient |
| Providing appropriate coverage for the patient |
| Taking the companions of other patients out of the room during intramuscular injection |
| Expelling non-mahram companions by pointing at the time of intramuscular injection |
| Taking the companions out of the room to respect the patient's privacy |
| Placing a screen for the patient |
| Closing the door of the room and taking out the companions during the foley cautery |
| Pulling the curtain while exposing the patient |
| Draw curtains between patients to protect privacy |
| Avoid unnecessary exposure of the patient |
| Avoiding undue exposure of the patient |
| Maintain patient information |
| Keeping patient information confidential |
| Not telling the patient's words to others if requested by the patient |
|  |
| **15- Respect the patient's beliefs and opinions** |
| Respect the patient by asking the patient's opinion |
| Respecting the patient who did not allow his beard to be cut by performing a bath in the stone |
| Respecting the patient's words by expressing dissatisfaction with being sent to another hospital |
| Respecting the patient's decision to undergo dialysis |
| Respect the patient's decision |
| Respect for the patient |
| Respecting the patient by transferring the patient's request to the doctor |
| Respecting the beliefs and cultural values of the patient |
| Respecting the patient by asking the patient's opinion |
| Respecting the patient's beliefs |
| Respecting the patient by paying attention to his needs |
| Fulfilling the patient's request and opinion |
| Fulfilling the patient's wish to have a male nurse |
| Consulting with the patient to take a vein |
| Giving the patient the right to choose as many veins as possible |
| Not forcing a non-psycho patient to perform procedures |
| Trying to get the patient's consent to perform the procedure |
| Protecting the patient's beliefs and values |
| Giving toilet bowls of the same gender to the same gender |
| Female nurse wearing gloves to take care of male patient |
| Wearing gloves when touching the pulse of a heterosexual patient |
| Adherence in venipuncture from the lower parts of female to male patients |
| Touching a non-homosexual patient with gloves as much as possible |
| Compliance with the law of gender compatibility with the patient as much as possible |
| Observing that the nurse and the patient are of the same sex |
| Compliance with the same gender of the nurse and the patient |
| Obtaining permission from the patient to perform the procedure |
| Obtaining permission from the patient to perform the procedures stated in the protocol |
| Obtaining permission from the patient to change the vein and give drugs at the same time |
| Getting permission from the patient to inject medicine |
| Obtaining the patient's permission to apply the cell pack |
| Obtaining permission from the patient by asking his opinion to perform the procedures |
| Obtaining permission from the patient to inject drugs for Covid-19 |
| Obtaining permission from the patient to inject expensive medicine |
| Obtaining the patient's consent to be admitted to the ICU |
| Obtaining consent before an invasive procedure |
| Allowing the patient to speak to express his needs |
|  |
| **16-Observance of justice** |
| Not discriminating between patients |
| Not discriminating between patients |
| Treating the patient with Covid-19 the same as his family members |
| The same treatment with conscious and unconscious patients |
| Equal treatment between patients |
| There is no difference between familiar and unfamiliar patients for the nurse |
| Expressing discomfort when the system discriminates against some patients |
| Being against allocating a bed to a patient known to the doctor |
| More treatment for Covid-19 patients than non-Covid-19 patients |
| Preventing the patient's rights from being violated in any way possible |
| Preventing the violation of the unconscious patient's rights by his son |
| Preventing violation of patient's rights by observing the scientific framework of care |
| Preventing the violation of the patient's right to be visited |
| Do not waste the right of the patient who is not familiar by not treating him badly |
| Reimbursement of the additional cost of the wrong drug in the system by the nurse |
| Failure to register the additional test fee for the patient due to laboratory error |
| Returning the discharged patient's medicine to reduce the cost |
| Making the right decision about the benefits and harms of the patient |
| Deciding to transfer to ICU for a patient who has more rights |
|  |
| **17- Sacrifice** |
| Working in the corona ward despite being pregnant |
| Coming to work despite having mental stress |
| The sacrifice of nurses provides moral care |
| The prominent spirit of self-sacrifice in nurses |
| Sacrifice nurses |
| Passing the shift despite having corona symptoms |
| Accepting the care of a Covid-19 patient despite the possibility of infection |
| Caring for a chronic patient despite having diabetes |
| It is not important for the nurse not to use the shield when the patient is in a bad condition |
| Teaching the patient despite the possibility of contracting the disease |
|  |
| **18- Self-control in difficulties** |
| Tolerating hardships in patient care |
| Tolerating the rigors of torturous disinfections |
| Tolerating the difficulty of wearing plastic clothing |
| Tolerating difficulty in meeting the patient's needs |
| Tolerating hardship in the care of commissioned patients |
| The hard tolerance of caring for a patient that suddenly expires |
| Tolerating washing and disinfection to reduce disease transmission |
| The great difficulty of nurses with corona coverage |
| Hardness of patient management for nurses |
| Difficulty giving oneself to obtain the patient's satisfaction |
| Being hard on yourself for doing the patient's work quickly |
| The difficulty of care during the long follow-up of the financial issues of the patient's drugs |
| Hospital care is more difficult than home care |
| Less rest in the night shift to meet the needs of the patient |
| It is not important to meet your own needs for the sake of meeting the needs of the patient |
| Carrying out responsibility despite the leave |
| Delaying one's physiological needs after meeting the patient's needs |
| Passing shifts throughout Nowruz Eid |
| Staying in the hospital longer than the required hours |
| Staying with the patient after hours is mandatory |
| Making yourself available until midnight to answer nurses' questions |
| Answering the phone even after the shift |
| Nurse working beyond the scope of her duties |
| Nurses working beyond their job description |
| Helping the patient after the end of the work shift |
| Overdoing work |
| Spending more time and energy than you can |
| Stopping to take the vein of a sick patient despite being stuck with a taxi service |
| Administering medicine instead of nurses by supervisors |
| Accepting the role of assistant or companion for the patient |
| Short-term tolerance of the conditions of the Corona sector |
| Difficulty controlling the nurse's feeling of concern towards the patient |
| Difficulty controlling the sad feeling of the death of the patients by the nurse |
| Nurses working with more patients during the peak of Corona |
| Multiple scrambles to schedule shifts |
| Repeated venipuncture for restless patient |
| The difficulty of nurses' work with heterosexual patients |
| Answers to repeated questions of patients |
| Answering the repeated questions of the patient with patience |
| Answers to repeated questions of companions up to 4 times |
| Answering the patient's constant and repetitive questions with reassurance |
| Be patient with the patient's frequent questions |
| Being patient in front of the patient's repeated calls |
| Making the nurse patient to listen to the patient |
| Emphasis on being patient |
| Being patient in critical and stressful situations |
| Increasing the work pressure of nurses during the holy month of Ramadan |
| Increasing the amount of care during the Corona era |
| Repeated dressing of a patient who opens his dressing |
| Working harder for patients with acute conditions of Covid-19 |
| Staying away from your children to reduce the possibility of disease transmission |
| The desire of the nurse to stay in hospital quarantine in order not to infect her family |
| Carrying out the resuscitation process with only two nurses |
| Management of patients with multiple problems |
| Management of patients and nurses in difficult conditions |
| Providing care with the simultaneous presence of multiple disorders in the patient |
| Providing care for all body organs in Covid-19 |
|  |
| **19-Patience** |
| Keeping silent in front of the patient's demanding behavior |
| Keeping silent in front of the rude patient |
| Keeping silent in the face of sick sarcasm |
| Keeping silent in front of the patient's unconventional behavior |
| Silence against tearing patients to pieces |
| Keeping silent in front of obscene patients |
| Do not raise your voice against the patient's repeated calls |
| Do not raise your voice in front of the patient |
| Do not raise your voice when asking the patient repeatedly |
| Do not shout at the patient |
| Nurses' tolerance towards aggressive movements of patients |
| The silence of the nurse in front of the cursing of the patient |
| Allowing the patient to accompany the patient for psychological evacuation |
| Staying with the patient despite hearing cursing |
| Leaving the place in front of the unconventional behavior of the patient |
| Leaving the room of the taunting patient |
| Do not argue with the patient |
| Do not growl at the patient who damaged his vein |
| Do not show anger |
| Not expressing annoyance from others verbally |
| Short coming against the inappropriate behavior of patients |
| Not getting nervous in front of insulting the patient |
|  |
| **20-Gentleness** |
| Speak kindly to the patient |
| Do not be violent with the patient |
| Be kind to the patient |
| Gentle treatment of the patient in the Covid-19 ward |
| Speak kindly to the patient |
| Apologize to the protesting patient |
| Add the word darling for respect |
| Calling young patients by their first or last name |
| Calling the patient by the patient's name |
| Addressing the patient by the name of Father Topol |
| Addressing younger patients by their first names |
| My dear father said to the elderly patient |
| Calling the patient by his last name |
| Calling the patient by his first name |
| Addressing the patient with a first name to increase intimacy |
| Dear father, address the patient |
| Addressing an elderly patient as father |
| Addressing the patient with the titles of father, mother, sister and grandfather |
| Dear father, address the patient |
| Calling the patient by his first name |
| Providing care with humor |
| Joking with an elderly patient by saying that you have colored eyes |
| Joke with the anxious patient |
| A joke about not finding a vein in a patient |
| Joke with the patient |
| A joke with a patient who was better |
|  |
| **21-Emotional compatibility** |
| Consoling the patient |
| Consoling the patient |
| Consoling the patient |
| Empathizing with the Covid-19 patient |
| Empathize with the patient's family |
| Empathizing with the patient by listening to the patient |
| Empathize with the patient |
| Having a state of empathy with the patient |
| Suffering the nurse while performing painful procedures for the patient |
| Listening to the patient |
|  |
| **22-Communicate with the patient** |
| Communicating verbally with the patient during procedures |
| Communicate by taking a brief history from the patient |
| Communicating properly with the patient as far as is physically possible |
| Communicating while closing the sphygmomanometer cuff |
| Communicating with the patient using sign language |
| Communicating with questions about the patient's personal information |
| Communicating better with the patient by being supported by a colleague |
| Communicating with the help of the content of the patient's words |
| Communicate by stating the reason for taking actions |
| Communication before the procedure |
| Communicating during care |
| Communicate well by explaining actions |
| Establish friendly communication by touching the patient |
| Establishing proper communication with the patient |
| Communicate with the patient as much as possible |
| Communicating with the patient during procedures |
| Communicating with the patient through writing |
| Communicate by touching the patient |
| Good communication with the patient |
| Communicate with the patient |
| Establishing proper communication with the patient during procedures |
| Communicate emotionally with the patient |
| Communicate by asking about the patient's current condition |
| Communication with the patient according to his mental and emotional conditions |
| Respectful communication with the patient |
| Communicate with the patient under the device |
| Try to communicate well with the patient |
| Talking to patients and explaining their treatment process |
| Talking to an unconscious patient |
| Talking with the patient during physiotherapy |
| Talking to the patient for a short time |
| Talking about everyday events with the patient |
| Talking to the patient like two friends |
| Introducing yourself to the patient |
| Introducing yourself to the patient |
| Introducing yourself to the patient |
| Introducing yourself to the patient |
| Introducing yourself to the patient |
| Introducing yourself at the beginning of communication with the patient |
| Maintain patient engagement through writing |
| Establishing more face-to-face interaction with the corona patient |
| Interact through touch |
| Waking up the patient to perform the procedure |
| Special greeting of the supervisor with the patients |
| Greeting patients during shift delivery |
| Greeting the patient at the beginning of the shift |
| Frequent visits to patients |
| Visit patients as much as possible |
| Frequent visits to the patient |
| Frequent visits to the patient |
| Frequent visits to patients with lower saturation |
| Using therapeutic communication |
|  |
| **23- Moral courage** |
| Not forcing the supervisor to do things that are useful for the patient |
| Not being strict in meeting the patient's rules |
| Dexamethasone drug injection based on the patient's condition |
| More serum injection than prescribed by the doctor |
| Apotel injection to a feverish patient without a doctor's order |
| Prescribing medicine by the nurse due to the shortness of the doctor |
| Using mobile phones in the ward for the benefit of the patient despite being prohibited |
| Opening the window of the ICU ward by the nurse for the benefit of the patient despite the ban |
| Increasing the sedation drug despite the doctor's order |
| Changing the doctor's prescription by the nurse |
| Do not put bi-pep against the doctor's order |
| Attempting to change treatment conditions for the benefit of the patient despite the doctor's order |
| Secret delivery of companions' food despite the doctor's orders |
| Allowing visits despite the blame of others |
| Allowing a companion to the ward after getting acquainted with the disease despite being prohibited |
| Permission to visit despite being forbidden |
| Allowing the family to visit the patient despite being prohibited |
| Allowing the companions of the patient to accompany the patient during the visit despite the ban |
| Allowing a companion to be with the patient despite quarantine rules |
| Permission to visit despite being forbidden |
| Allowing the visit of the patient and companions despite the ban on the presence of companions |
| Allowing the family to stay with the patient longer, despite the legal restrictions |
| Changing the patient's breathing mask despite the doctor's order |
| Changing the patient's mask and informing the doctor |
| Putting serum on a fasting patient without a doctor's order |
| Adjusting the best setting of the ventilator despite the doctor's order |
| Taking actions based on the patient's benefit despite the doctor's order |
| Doing the right thing despite the blame of others |
| Doing the right thing regardless of what others think |
| Doing the right thing even secretly |
| Not fulfilling the inappropriate request of the patient |
| Arguing with the head of the ward about placing an order for the residents before the patient's visit |
| Objecting to the doctor who did not respect the patient's opinion |
| The courage to object to absenteeism from colleagues |
| Inner disgust of doing work without principles |
| Dealing with an incompatible companion of the patient |
| Dealing with the crew's irrational behavior with the patient |
| Not allowing impatient patient companions to the hospital |
| Not allowing the company that disturbs the peace of the patient |
| Hanging up the phone when hearing inappropriate words from the supervisor |
| Doing better without a doctor's order based on experience |
| Giving medicine without a doctor's prescription |
| Changing the mask to improve saturation before the doctor's order |
| Changing the patient's mask depending on his condition and before the doctor's order |
| Giving medicine for the patient's constipation without a doctor's order |
| Prescribing effective medicine without a doctor's prescription |
| Lasix injection to a patient with edema without a doctor's order |
| Pantazol injection without a doctor's prescription |
| Injection of anti-allergic medicine without a doctor's prescription |
| Injection of medicine to reduce the side effects of corona medicine without a doctor's order |
| Injection of medicine before the doctor's order |
| Carrying out care for the benefit of the patient without a doctor's order |
|  |
| **24- Internalized values of the nurse** |
| Granting the right to the patient to know the progress of the disease |
| Considering the patient's right to consent in performing the procedure |
| Granting the right to know the length of hospitalization |
| Granting the right to know about the treatment process |
| Granting the right to information about medicines |
| Granting the right to more care for the patient |
| Granting the right to answer the patient's questions |
| Asserting the right to have a companion |
| Asserting the right to eat hot food |
| Granting the patient the right to contact the family |
| Giving rights to the nurse and the patient |
| Considering the right to be upset for patients |
| Giving the patient the right to visit on time |
| Giving the patient the right to know |
| Giving the patient the right to know |
| Granting the right to receive the things that the companions bring for the patient |
| Admitting the right to separate hospitalization for male and female patients |
| Granting the patient the right to receive free treatment |
| Giving the patient the right to choose |
| Granting the right to change the doctor for the patient |
| Assuming the right not to worry about money for the patient |
| Granting the patient the right to know the time of the visit |
| Granting the right to know about medicines |
| Admitting the right to have enough space to admit the patient |
| The importance of gender differences in admitting patients for nurses |
| The importance of having the patient's words for the nurse |
| The importance of praying in any situation for nurses |
| The importance of the elderly patient being sensitive to hijab for the nurse |
| The importance of having the mental aspect of the patient as well as the physical aspect for the nurse |
| The importance of having the mental aspect of the patient for the nurse |
| The importance of patient coverage for nurses |
| The importance of patient coverage for nurses |
| The importance of promoting the nursing profession |
| The importance of the patient's comfort for the nurse |
| The importance of income for nurses |
| The importance of individual beliefs for nurses |
| The importance of giving medication on time for nurses |
| The importance of having the patient's words for the nurse |
| The importance of patient physiotherapy for nurses |
| The importance of having a drug injection at a suitable speed for the nurse |
| The importance of respecting people's rights for nurses |
| The importance of patient nutrition for nurses |
| The importance of having the patient eat for the nurse |
| Not allowing the nurse to stop drugs intentionally |
| It is human ignorance to give bad news to the patient |
| Considering a chronic patient as a person with understanding and feelings |
| Considering the Covid-19 patient as a human being |
| Expecting financial support to work in the Corona sector |
| Getting strength from God to work with corona patients despite the illness |
| Believing in honesty and integrity |
| Believes in seeing the rewards of actions in this world |
| Believing in not angering the patient |
| The importance of working conscientiously |
| The importance of working conscientiously |
| The importance of providing conscientious care |
| The importance of acting conscientiously in not doing less for the patient |
| The importance of having a conscience for nurses |
| Painful conscience by not giving medicine to the patient |
| Conscientiousness by not doing better for the patient |
| Conscience is bothered by not doing moral care |
| A twinge of conscience while not doing things according to principles |
| The concern of conscience in receiving a salary commensurate with the work |
| Compulsion of conscience to replace non-sterile infusion sets with burettes |
| Conscience is effective on the quality of work |
| Conscience is effective in providing ethical care |
| Conscience is effective in providing better care |
| Knowing conscience to be effective in pursuing more to find a rare drug |
| Considering the patient's body as dirty by nurses |
|  |
| **25- Evidence-based experiences of nurses** |
| Attention to the effect of the patient's relaxation on the blood saturation level |
| Attention to the effect of anxiety on patient non-cooperation |
| Paying attention to the effect of disease anxiety on the treatment process |
| Attention to the effect of anxiety on the deterioration of the patient's condition |
| Attention to the effect of stress on the deterioration of the treatment process |
| Attention to the effect of stress on the deterioration of the patient's disease process |
| Attention to the effect of stress on the worsening of disease symptoms |
| Attention to the effect of stress on the worsening of the patient's condition |
| Paying attention to the effect of stress on bi-pep intolerance |
| Attention to the effect of stress on the patient |
| Paying attention to the effect of stress on the patient's erection |
| Attention to the impact of stress caused by the death of loved ones on the patient's recovery |
| Paying attention to the effect of being active on helping to improve the disease process |
| Paying attention to the effect of activity on improving the patient's treatment process |
| Attention to the effect of psychological support on the patient's condition |
| Paying attention to the effect of praying on increasing the hope of healing in patients |
| Attention to the effect of the patient's good mood on the treatment process |
| Paying attention to the effect of the difficulty of tolerating bi-pep on stress |
| Attention to the effect of having shortness of breath on increasing stress in patients |
| Paying attention to the effect of the patient's realization of low saturation on stress |
| Paying attention to the effect of prayer on the patient's comfort |
| Paying attention to the effect of seeing the death of the side bed on creating fear |
| Attention to the effect of staying with the patient on reducing stress |
| Paying attention to the effect of the nurse's presence next to the patient on increasing saturation |
| Attention to the effect of relaxation on reducing distress |
| Attention to the effect of relaxation on increasing saturation |
| Paying attention to the effect of talking to God on the patient's comfort |
| Paying attention to the effect of knowing about the disease on increasing the cooperation of the patient |
| Attention to the effect of positive thinking on reducing respiratory distress |
| Paying attention to the effect of compulsion to tell the price of medicines on increasing the patient's stress |
| Attention to the effect of obesity on the incidence of worse prognosis in patients |
| Attention to the effect of hypoxia on the patient's moodiness |
| Paying attention to the direct effect of low saturation on patient expiration |
| Paying attention to the direct effect of low level of consciousness on the expiration of the patient |
| Paying attention to the effect of losing morale on the patient's expiration |
| Paying attention to the effect of the nurse's frustration on inappropriate communication with the patient |
| Paying attention to the effect of income on motivating nurses |
| Attention to the effect of money and material issues in providing moral care |
| Attention to the impact of financial well-being on the quality of care |
| Paying attention to the effect of economic conditions on establishing proper communication with colleagues |
| Paying attention to the effect of a better economic situation on establishing a proper relationship with the patient |
| Attention to the influence of elders and veterans in the field of service |
| Paying attention to the effect of the clergy's role in reducing the patient's stress |
| Paying attention to the effect of personal habits and nurse training in relation to the patient |
| Attention to the influence of the nurse's lifestyle in providing ethical care |
| Attention to the patient's severe dependence on oxygen |
| Paying attention to the sensitivity of the Covid-19 patient to the reduction of arterial oxygen |
| Paying attention to the patient's smell and taste disorders |
| Paying attention to the time limit in eating for a patient who has a rapid decrease in saturation |
| Paying attention to the rapid change of saturation level in the patient |
| Attention to the development of drug resistance in the patient over time |
| Paying attention to the occurrence of black fungus in patients receiving corticosteroids |
| Paying attention to unnecessary expenses for the patient |
| Paying attention to the unnecessary admission of the patient to the hospital |
| Paying attention to admitting the patient without reason |
| Paying attention to writing unnecessary advice and referrals |
| Paying attention to the prescription of judges that the patient does not need |
| Attention to unnecessary prescription of Favipiravir tablets |
| Attention to unnecessary Remdesivir injection |
| Attention to the unnecessary injection of Remdesivir to end-stage patients |
| Attention to the unnecessary injection of Remdesivir after the viral phase |
| Attention to the prescription of unnecessary drugs |
| Paying attention to the unnecessary conduct of some tests on a daily basis |
| Perceiving the patient's reluctance to communicate during the patient's statements |
| Perception of the patient's reluctance to insert Foley |
| Perception of the patient's reluctance to perform CT angiography |
| Perception of making the patient feel good by asking his opinion |
| Perception of making the patient feel good about taking corona drugs |
| The perception of creating a good feeling in the patient with nurses' sense of humor |
| The perception of creating a sense of hopelessness in the patient when there is no communication |
| Perception of the patient's sense of hopelessness towards recovery |
| Understanding the feeling of hopelessness in patients with patient statements |
| Understanding the feeling of discomfort in a patient who is not allowed to approach the nursing station |
| Understanding the patient's feeling of being upset about not leaving enough time for him |
| The perception of creating a sense of shame in the patient with the patient's statements |
| Perceiving the feeling of losing morale in the patient when the patient does not communicate |
| Perceiving the patient's sense of comfort towards the nurse from the patient's tone |
| Perceiving the feeling of angering the patient with life by expressing the desire to die |
| Realizing the existence of the patient's sense of trust in the nurse with the request for the nurse to come again |
| Understanding the sensitivity of the patient for further care |
| Perceiving the possibility of the patient feeling ashamed of his illness |
| The perception of anxiety in the patient regarding the treatment process |
| The perception of anxiety in the patient regarding the treatment process |
| The perception of the presence of stress towards death in the patient |
| The perception of the presence of stress in the patient in relation to the corona virus |
| The perception of the presence of fear and terror towards death in the patient |
| Perception of fear of death in the patient |
| Perception of fear of death in the patient |
| Perception of fear of death in the patient |
|  |
| **26- Nurse tendencies and instincts** |
| Being a joker |
| Being a joker |
| Being a joker |
| Being follower of issues |
| Being patient |
| Being curious |
| Studying and its impact on the provision of informed care |
| Criticizing |
| Easy to communicate with others |
| Doing things quickly |
| Being meticulous at work |
| Being sensitive to relationships with colleagues |
| Loving the nursing job |
| Loving the nursing job |
| Having a good sense of nursing |
| Loving the nursing |
| Loving the ward that works |
| Enthusiasm and interest in work |
| Being interested in patients of the same age |
| Interest in learning scientific material |
| Interested in studying |
| Interest in knowing and raising awareness |
| Interest in learning more due to interest in nursing profession |
| Interested in saving one's health by opening the windows while taking a corona test |
| Interested in maintaining one's health by reducing the frequency of unnecessary contact with the patient |
| Interested in maintaining one's health by reducing the frequency of unnecessary contact with the patient |
| Maintaining one's health by reducing your exposure to Corona by changing the number of people in the recovery protocol |
| Interested in maintaining one's health by using special corona coverage |
| Interested in maintaining one's health with more personal protection in times of fear of Corona |
| Interested in maintaining one's health by providing care with more personal protection |
| Interested in maintaining one's health by using shield, gun and gloves to take the test |
| Interested in maintaining one's health by using special coverage in the Corona section |
| Nurses are dependent on each other |
| Being hot-tempered |
| Nurse's stress and its effect on care |
|  |
| **27- The mental suffering of the nurse** |
| Involvement of the nurse's thoughts on the patient who died |
| Constant thinking of the nurse to the patient even at home |
| Thinking of the nurse to the patient even at home |
| Constant thinking of the nurse to the patient |
| Keeping the patient's memories in the nurse's mind for a long time |
| Keeping the patient with coronary heart disease in the nurse's mind |
| Uncomfortable scenes remain in the nurse's mind |
| The sad memories of several patients remain in the nurse's mind |
| The mental concern of the nurse to implement a better treatment method |
| Making mistakes in writing reports when the nurse is upset with a person |
|  |
| **28- Emotional suffering of the nurse** |
| Having the mental concern of transferring corona virus to the family from the nurse's side |
| The nurse's concern about the family's infection with Covid-19 |
| Diabetic nurse's concern about own’s infection with Covid-19 |
| A feeling of insecurity from the authorities |
| Unhappiness with the presence of authoritarian rule in the ward |
| Sadness of not meeting the educational needs of a diabetic patient with corona |
| Discomfort from not injecting medicine to the patient by some of the colleagues |
| Discomfort from colleagues who do not use sterilization methods before catheterization |
| Discomfort from not being able to do the correct sampling which caused financial loss |
| Discomfort with not injecting the patient's medicine by some colleagues |
| Discomfort from pulling the peripheral venous catheter without wetting the glue by some nurses |
| Discomfort from working in the hospital |
| The discomfort of multiple patient expirations for nurses |
| Getting upset with the patient's companions |
| Discomfort and depression of nurses from expiring patients |
| Depressing nurses by frequently seeing patients waiting to die |
| Damage to the morale of nurses by observing many deaths |
| Damage to the morale of nurses |
| Damage to the morale of nurses with the death of patients who were hospitalized for a long time |
| The nurse's condition worsened |
| Bad mental condition of the nurse |
| Mental and psychological harassment of the nurse during forced intubation of the patient |
| Tears of the nurse during the CPR of her patient |
| Crying nurse at the death of a young mother |
| Crying for a respected deceased patient |
| The nurse's unhappy caused the patient's needs to be met late |
| Not having a good feeling for a chronic cancer patient in the nurse |
| Dare not to object to the doctor's words |
| The courage not to protest in front of the supervisor |
| Courage not to object to the inappropriate behavior of the supervisor |
| Courage not to protest wrong prescriptions |
| Forced to teach the patient secretly while being mocked |
| Forced to secretly do things contrary to the opinion of the patient or the hospital |
| Forced to secretly do ethical work |
| Forced to blend in with the crowd |
| Forced not to reveal religious sensitivities to the shift manager |
| Lowering the threshold of tolerance in nurses |
| Occurrence of discomfort in the nurse in times of poor cooperation of some nurses |
| Occurrence of anxiety in nurses in times of poor cooperation between colleagues |
| Occurrence of anxiety in times of tension between colleagues |
| Corona virus stress in nurses |
| The presence of fear in nurses from the name of corona disease |
| The stress of the family's illness caused her to leave the nursing |
| The stress of being infected with Corona caused her to quit nursing |
| The stress of passing the disease on to the family |
| The nurse's stress from her husband's infection with Covid-19 |
| The nurse's stress from infecting the family |
| The nurse's stress about the family's infection with Covid-19 |
| The stress of getting infected with corona in nurses |
| The nurse's stress from infected with corona disease |
| Stress in nurses |
| Having stress when caring for Covid-19 patients |
| Fear of Covid-19 prevents communication with the patient |
| Fear of Covid-19 in nurses |
| The frown of the nurse and its effect on the lack of proper communication |
|  |
| **29- Social suffering in providing care** |
| The stigma of being overly sensitive to the nurse following the patient's affairs |
| Objected by colleagues about not being violent with the patient |
| Stigmatization of illegal informant nurse |
| Making fun of the nurse for wearing gloves |
| Threating nurse by the supervisor |
| Bullying a newly arrived nurse |
| Objected by nurses to heating the patient's food in the nurses' room |
| Not allowing nurses to use their vacation rights |
| Reprimanding the nurse when she protests |
| Inappropriate handling of the nurse's protest |
| Making the working conditions of protesting nurses more difficult |
| Weak support from officials to employees |
| Changing the objectionable supervisor to doctors |
| Not asking the nurse to move the ward that works |
| Compulsory sending of nurses to the corona ward |
| Ignoring the efforts of the nurse |
| Ignoring personal problems of nurses |
| Injustice in assigning work shifts for nurses |
| Not understanding the nurses by the authorities |
| Failure to fulfill financial promises to the nurse |
| Inadequate appreciation of nurses |
| Not appreciating the efforts of nurses |
| Not appreciating the authorities |
| Not giving proper appreciation to the nurse of the corona ward |
| Inappropriate behavior with supervisor when not accepting the leave of nurses |
| Not valuing the nurse |
| Not valuing the nurse |
| Not valuing a nurse who does a good job |
| Lack of trust and value to nurses from society |
| Compulsion to perform tasks beyond the job description |
| Mistreatment of nurses by supervisor |
| Injustice and coercion of colleagues and supervisors |
| The words of an experienced nurse are not important for a doctor |
|  |
| **30- Legal restrictions** |
| Changing the CPR protocol prevents the implementation of CPR according to the legal time |
| Hospital policies hinder the provision of timely care |
| Laws prevent the implementation of ethical work |
| Time-consuming control of vital signs in 4 times |
| The time-consuming process of admitting a Covid-19 patient |
| The doctor disagrees with the correct decisions of the nurse |
| Lack of clarity of priority in the doctor's order or hospital policy |
| Forced to carry out drug orders whose effect was not known |
| Compulsion to take a flat line ECG and record it in the case of failed resuscitation |
| Compulsion in accordance with the law to obtain personal consent from patients for discharge |
| Compulsion to follow the doctor's order |
| Compulsion to administer medicine in the hospital |
| Forcing the patient to pay for the medicine even if the nurse does not inject it |
| Compulsion to inject medicine after the patient has paid the money |
| Compulsion to inject Remdesivir in abnormal saline serum |
| Compulsion to prescribe Remdesivir based on the ward's routine |
| Forced to ineffective prescriptions |
| Compulsion to act on contradictory medical orders |
| Forced to inject corona drugs, which the nurse does not agree with |
| Forcing a sedative injection against the doctor's orders |
| The compulsion to complete each room causes overcrowding of patients |
| Compulsion to inject dexamethasone based on the ward's routine |
| Rules that waste the nurse's time and energy |
| A lot of written work hinders communication |
|  |
| **31- Structural constraints** |
| Shortage of oxygen |
| Serum deficiency in the ward |
| Low serum count |
| Serum deficiency |
| Shortage of paravan in the ward |
| Paravon deficiency |
| Shortage of clothes for the patient |
| Shortage of advanced equipment |
| Shortage of equipment and facilities hinders the provision of correct and timely care |
| Shortage of equipment |
| Shortage of equipment hinders the provision of ethical care |
| Shortage of microwave to heat food for patients |
| Shortage of equipment and facilities in the ward |
| Shortage of hospital facilities hinders the patient's privacy |
| Shortage of beds causes rejection of the patient's bed transfer request |
| Shortage of equipment prevents the provision of proper care |
| Shortage of medicine causes the accumulation of nurses' duties for the next shift |
| Shortage of facilities to heat the patient's room |
| Shortage of hospital space |
| Shortage of oxygen with increasing number of patients |
| Shortage of extra blanket to cover the patient |
| Shortage of healthy pulse oximetry to check blood oxygen |
| The effect of hospital management on care delivery |
|  |
| **32- Shortage of nurse** |
| Shortage of nurses in the ward |
| Shortage of nurses in the ward |
| Shortage of male nurses |
| Shortage of nurses when there are many sick people |
| Shortage of nurses when nurses are sick |
| Shortage of nurses |
| Shortage of nurses |
| Shortage of nurses prevents proper care of the patient |
| Shortage of nurses prevents the provision of complete care |
| Shortage of number of nurses hinders ethical care |
| Shortage of nurses prevents the non-medical needs of the patient from being met |
| Shortage of nurses hinders compliance with the compliance plan |
| Shortage of service personnel |
| Shortage of nurses causes non-observance of gender compatibility law |
| Shortage of nurses causes not responding to the patient's request |
| Shortage of nurses causes insufficient attention to the patient |
| Shortage of nurses hinders the provision of adequate care |
| Shortage of nurses |
| Shortage of nurses causes a lack of complete and ethical care for the patient |
| Shortage of nurses makes it impossible to meet all the needs of the patient |
| Shortage of nurse's help causes the patient's food not to be given on time |
| Non-standard number of nurses compared to patients |
| Resignation of nurses due to family problems |
| A large number of shifts and mental pressure hinder the provision of ethical care |
| The number of shifts and more overtime hinders the provision of ethical care |
| A large number of shifts hinders better and more ethical treatment of the patient |
| A large number of shifts reduces communication with the patient |
| The number of shifts causes nurses to be tired |
| The number of shifts causes fatigue |
| The large number of patients hinders more time to treat each patient |
| The large number of patients prevents more communication with patients |
| The large number of sick patients hinders the provision of better care |
| The large number of patients causes a decrease in responsiveness to the patient and companion |
| The large number of patients increases the workload |
| Not having enough time prevents further treatment of the patient |
| Not having enough time to check the patient's blood sugar every 6 hours |
| Time constraints, preventing more time for each patient |
| Shortage of time prevents blood sugar control at the prescribed times |
| Shortage of time prevents monitoring of vital signs as ordered |
| Shortage of time causes patients to prioritize their work |
| Shortage of time prevents the delivery of the equipment that the patients' companions bring to the patients |
| Not having the opportunity for the nurse to make eye contact with the patient |
| Not having the opportunity to educate the patient |
| High workload hinders the nurse's accuracy |
| High workload causes lack of time |
| High workload causes the error of not doing the work |
| High workload increases the occurrence of errors |
| High workload causes errors |
| High workload causes not talking to the patient enough |
| High workload causes the doctor's order not to be implemented accurately |
| Less workload makes it easier to provide ethical care to patients |
| Carrying out a lot of necessary care for a patient |
| Carrying out a lot of necessary care for the patient |
| Busy work causes the wrong injection of medicine |
| Crowding prevents the patient from calming down |
| Overcrowding in the ward causes the medicine not to be given |
| Overcrowding causes neglect of the patient |
| Overcrowding makes the nurses tired and sleep in the nursing station |
| Congestion affects the nurse's behavior |
| Overcrowding in the hospital reduces interest in nursing |
|  |
| **33- Reducing the suffering of nurses** |
| Trusting the doctor to the trusted nurse |
| Appreciate the nurse with a bouquet of flowers |
| The patient's gratitude to the nurse |
| Appreciating nurses |
| Taking nurses to the theater for free |
| Getting a nurse's day celebration in the ward |
| Calling ward nurses when necessary |
| Trying to reduce the difficult mental conditions of the nurses in the corona ward |
| Taking the lead in doing things |
| Accepting leave requests of nurses as much as possible |
| Not bolding the reaction of nurses to high work pressure |
| Positively considering the reaction of nurses despite the heavy workload |
| Having a positive view of nurses and influencing their performance |
| Lack of up-down vision |
| Not misinterpreting nurses' reaction to high work pressure |
| Covering work shifts based on nurses' conditions and experience |
| Writing the holiday program so that the nurses are off for a few days |
| Balancing nurses' shifts during the peak of Corona |
| Establishing justice in the division of overworked patients among nurses to solve problems |
| Fair distribution of shifts based on experience |
| Fair distribution of critical patients among nurses |
| Division of work based on experience and insight into each person's situation |
| Division of work according to the mental, psychological and physical conditions of nurses |
| Enhancing employee insight to perform work without manager supervision |
| Improving the morale of the nurse with the good prayer of the patient |
| Improving the morale of nurses by paying attention to them |
| Explaining to nurses to improve their understanding of doing work |
| Be friendly with the ward nurses |
| Nurses support each other |
| Doing things in a friendly way, not in an orderly manner |
| Empathizing with colleagues to pass the shifts |
| Try to understand the problems of colleagues by putting yourself in the place of colleagues |
| Trying to prevent colleagues from hiding |
| Reminding nurses about how to dress with humor |
| Asking the problems of nurses from the nurses themselves |
| Relative success in resolving conflict between employees |
| Managing conflicts between colleagues by giving incentives |
| Fair management of supervisors to resolve conflicts between nurses |
| Interaction with nurses whose morale was affected by the death of the patient |
| The effect of management support in providing ethical care |
| Talking with nurses to solve work problems and reduce stress |
| Talking to calm the nurses |
| Changing the resting place of sick nurses |
| Providing a place to rest for nurses who are worried about the transmission of the virus |
| Providing another place to rest the personnel who had the stress of contracting Corona |
| Providing another place to sleep for personnel who have corona symptoms |
| Providing conditions for nurses to talk directly with psychologists |
| Providing an error reporting system and error expression to gain experience |
| Assessment of mental health of nurses |
| Referral of new forces to a psychologist for mental health assessment |
| Sending nurses to psychology classes |
| Creating a Telegram application group with the presence of a psychiatrist to vent emotions |
| Giving leave to nurses who tested positive for Covid-19 |
| Transferring problems of nurses to officials |
| Transferring a nurse with diabetes to a less dangerous ward |
| Transferring the protesting nurse to the non-corona ward |
| Transferring nurses who had problems to non-coronavirus wards |
| Allow time to train new nurses |
| Giving information to nurses about new medical and care cases |
| Request to the director of the hospital for the presence of more clergy |
| Placing a hanger to hang the clothes of the corona section before resting |
| Prioritizing the nurses of the ICU ward in getting masks |
| Efforts to provide protective equipment |
| Trying to prepare detergent for the ward |
| Trying to change the spirit of ward nurses |
| Giving incentives to special ward nurses |
| Giving off incentives for more productive nurses |
| The supervisor's frequent visits are a kind of support for the officials in providing nursing care |
| Promising words from officials |
| Officials give hope to nurses and improve their morale |
| Encouragement from the shift manager for observing ethical points |
| Showing care for nurses even with little financial support |
| Giving money to nurses for hardship during the corona |
| Traveling with hospital money for a nurse working in the corona ward |
| Implementation of accreditation rules in the hospital |
| Caregiver support in providing ethical care |
| Officials' support for nurses in the form of effective payment of Corona |
| Financial support for nurses during the Corona |
| Supporting the nursing office to provide ethical care |
| Physicians' support for nurses' decisions and actions |
| Supporting the doctor to the nurse in front of the patient |
| The doctor's support for the nurse's decision regarding oxygen supply to the patient |
|  |
| **34- Compensating for equipment and nurse shortages** |
| Maintaining an empty bed by Hourly hospitalization of the patients |
| Hourly hospitalization of the patient for drug injection |
| Using a clinic instead of a ward for hourly hospitalization of patients |
| Supplying shortage equipment from other wards |
| Solving the shortage of stretchers by requesting other wards |
| Mask preparation at personal expense |
| Using two simple masks and handkerchiefs when there is a shortage of N95 masks |
| Using expired serum in times of serum shortage |
| Using one serum for several patients in times of serum shortage |
| Keeping serum after three days in case of serum deficiency |
| Covering the shirt without pants when there is a lack of clothes |
| Using oxygen capsules instead of central oxygen in emergency situations |
| Adding a new bed in the doctor's room |
| Admitting an isolated patient in a room with two beds when there is no isolation room |
| Continuous patient monitoring during oxygen device failure |
| Stratification of patients for hospitalization for Remdesivir injection |
| Prioritizing patients for better treatment of patients |
| Carrying out patients' work despite the shortage of equipment and beds |
| Continuous patient monitor in times of equipment shortage |
| Rotating the hospitalization of patients in different wards |
| Transfer of auxiliary staff from other hospitals |
| Getting help from other hospitals |
| Power supply from closed sectors |
| Getting help from non-coronavirus wards |
| Getting help from other wards |
| Contract extension to compensate for the shortage of nurses |
| Getting help from the supervisor to increase the number of nurses in the ICU ward |
| Planning to hire contract nurses |
| Managing the shortage of nurses by promising nurses less shifts and more leave |
| Ward management with few nurses |
| Reducing the work pressure of nurses by creating a new ward |
| Reducing the work pressure of nurses with the arrival of jihadist groups |
| Reducing the workload of nurses by not accepting non-coronavirus patients |
| Reduce workload by receiving weekly or monthly medication instead of daily |
|  |
| **35- Helping colleagues in providing care** |
| Helping and being friends with colleagues |
| Help the nurses by taking a vein from a patient with a bad vein |
| Helping a colleague to get IV |
| Helping colleagues to do patients' work |
| Helping the nurse by helping her sick patient |
| Getting help from colleagues to provide scientific care |
| Colleagues helping each other encourage work |
| Helping nurses to each other in performing their duties |
| Sympathetic cooperation between colleagues prevents mistakes |
| Cooperation between nurses reduces stress |
| The cooperation of nurses in coming shifts instead of each other |
| Cooperation between nurses and its effect on patient care |
| Cooperation between nurses to come to each other's shifts |
| Cooperation of nurses in completing the shift schedule |
| Cooperation and correct communication between colleagues and its effect on patient care |
| The cooperation of nurses to create a balance of nurses in the ward |
| Neutralizing between colleagues to create peace |
| Entrusting the patient's venipuncture to the most skilled nurse in the ward |
| Leaving patients to colleagues when going for prayer |
| Handing the patient over to colleagues to take care of another patient |
| Entrusting the Stat dose of the patient's medicine to colleagues |
| Consult with experienced people and hospital officials about the rules |
| Consult the doctor to take the correct action for the patient |
| Consultation with the doctor to create better conditions for the patient |
| Consultation with the doctor and analysis of the patient's condition to resolve the ambiguity and make a decision |
| Creating the necessary context for the patient's companions to consult with the doctor |
| Ask the doctor to clear the confusion |
| Asking experienced colleagues to clear care doubts |
| Newbie nurses' questions to experienced nurses to prevent mistakes |
| Asking the doctor about the patient's request to leave the ward |
| Asking the doctor to convince the patient not to be discharged |
| Reminding the doctor for a patient visit |
| Reminding the doctor to place a prescription |
| Reminding the doctor to change the order of the patient's mask type |
| Reminding the doctor to reduce the dose of medicine |
| Reminding the doctor to correct the wrong prescription |
| Reminder of the side effects of Haloperidol by installing a pamphlet on the board of the ward |
| Advice to colleagues to choose the best mask |
| Advice to the doctor to prescribe better medicine |
| Advice to the doctor not to transfer the patient |
| Notice to the doctor about correcting the prescription |
| Verbal warning to medical residents about side effects of prescription drugs |
| Warning to medical residents by taking minutes to prevent Haloperidol side effects |
| Tolerating nurses with each other |
| Mutual understanding between colleagues |
| The interaction of the treatment team to find a solution for the improvement of patients |
| Interaction between nurse and doctor to improve the patient's condition |
| Interaction between nurse and doctor for the benefit of the patient |
| Interaction with ICU resident nurse to transfer more needy patient to ICU |
| Collaborative decision making in times of uncertainty |
| Team participation to convince the patient to stay in the hospital |
| Improving the quality of care for teamwork |
| Performing basic procedures through group work |
| Doing teamwork by creating a friendly atmosphere among nurses |
