# Supplementary material for: Ethical care in patients with Covid-19: A grounded theory
Source: PLoS One. 2024 Mar 28;19(3):e0300156. doi: 10.1371/journal.pone.0300156 (PMC10977892; doi:10.1371/journal.pone.0300156)
Supplement: Attachment — Submitted filename: Response to Reviewers.docx [file pone.0300156.s003.docx]

**In The Name of God**

**Hello Dr. Myriam**

**The reviewers' excitement for the article and the insightful criticism they and the Chief Editor provided are both sincerely appreciated. The manuscript's readability and validity have been greatly improved by these observations.**

**I edited the article based on the reviewer's comments. Also I responded to all comments.**

**Please see the table below.**

**Gratitude once more.**

| **Chief editor comments** | **Author's response** |
| --- | --- |
| Please state what role the funders took in the study. If the funders had no role, please state: "The funders had no role in study design, data collection and analysis, decision to publish, or preparation of the manuscript." | Was corrected. (Written in the manuscript with blue highlights) |
| Please include this amended Role of Funder statement in your cover letter; we will change the online submission form on your behalf. | This statement was written in cover letter and highlighted in blue. |
| In your Data Availability statement, you have not specified where the minimal data set underlying the results described in your manuscript can be found. PLOS defines a study's minimal data set as the underlying data used to reach the conclusions drawn in the manuscript and any additional data required to replicate the reported study findings in their entirety. All PLOS journals require that the minimal data set be made fully available. pon re-submitting your revised manuscript, please upload your study’s minimal underlying data set as either Supporting Information files or to a stable, public repository and include the relevant URLs, DOIs, or accession numbers within your revised cover letter. | The DOCX file of all primary codes was uploaded as a S1 File. Access to underlying information is written in blue highlighter in the Ethical Considerations section. |
| PLOS requires an ORCID iD for the corresponding author in Editorial Manager on papers submitted after December 6th, 2016. Please ensure that you have an ORCID iD and that it is validated in Editorial Manager. To do this, go to ‘Update my Information’ (in the upper left-hand corner of the main menu), and click on the Fetch/Validate link next to the ORCID field. This will take you to the ORCID site and allow you to create a new iD or authenticate a pre-existing iD in Editorial Manager. | Was corrected. |
|  |  |
| **Reviewer 1** | **Author's response** |
| There is a need for clarification about the recruitment procedure, by whom? | By the first author (Written in the method section with blue highlights) |
| Please clarify theoretical sampling and saturation all readers are not familiar with these terms. | The explanation of theoretical sampling and theoretical saturation was written in the method section with blue highlights. |
| Interview guide revised after every interview? It should be after every analysis—that is when you know if there is something new to focus on. | This is what the author meant, that is, the editing of the interview questions is done after the analysis of each interview. Corrections are highlighted in blue. |
| Information about the interview guide? And pilot interviewing was it performed? Please, clarify and give us some more information. | The first interview was conducted as a pilot and after its analytical review by the research team, more appropriate questions for the next interview guide were developed. The first version of the interview guide is available within the manuscript (S1 Table). Corrections are highlighted in blue. |
| How come that there were 21 interviews, but 19 participants? | In order to complete the categories and clarify some ambiguities in the interviews, two participants were interviewed twice. Written in method section. Corrections are highlighted in blue. |
| After 19 interviews there was no new data, meaning that the categories were filled and stable?? No new codes or categories were identified. | Theoretical saturation of categories occurred when no new category was obtained during data coding. It should be noted that no new category was obtained in the last two interviews. In fact, after 19 interviews, we reached theoretical saturation. But to ensure theoretical saturation, two more interviews were also conducted. Finally, 21 interviews were conducted. Corrections are highlighted in blue. |
| There was a single researcher performing the interviews, how did this person manage to make observations and taking notes at the same time? | The first author used the unstructured non-participative observation technique during the interview, so that she recorded the important things in a brief and headlined manner, then immediately after the interview and leaving the hospital, she added more details to it. Therefore, there was no need for a second person. These items were written in the text with blue highlights. |
| If taking/spending shift as a nurse it is hard to do observations. Were you there as an observer? | The observation of the researcher was in an unstructured way, that is, the researcher wrote down the things that seemed important to her in the form of headings in order to add more details to them at the right time. On the other hand, the researcher was present in the hospital as a nurse's assistant, so she had more freedom in this field. |
| The analysis is presented according to the book. However, there was no axial coding? | The analysis was based on Corbin and Strauss 2008. According to it, there should be 4 stages of data analysis for concepts, data analysis for context, data analysis for process, and integration of classes and presentation of theoretical structure. These 4 steps are explained in the method section. It is highlighted in blue. |
| Trustworthiness is presented, according to reference 27. Grounded theory do not us these concepts (since it does not present itself as an ordinary qualitative method)- since it has its own quality criteria. Corbin and Strauss present 8 criteria. Strauss and Corbin do clarify how credibility, trust, and applicability etc are to be managed in GT. | Was corrected. Rigor and trustworthiness were written according to Corbin and Strauss criteria and highlighted in blue in table 1. |
| The result- information about the participants can be placed in the methodology section—sample (free of choice). | Some information such as the number of interviews and the number of participants were written in the methodology section and removed from the results section. |
| Decide if the information should be in text or in table, now it is duplicated. | Duplicate content in the text was removed. |
| Still, confusion about 21 interviews and only 19 participants. | Two participants were interviewed twice. In order to complete the categories and clarify some ambiguities in the interviews, two participants were interviewed twice. |
| The result presentation would benefit from be presented below table 1. | The information related to the table (The characteristics of the participants) was written below it. It is highlighted in blue. |
| In total 10…… bridging over to the presentation: Main concern The result presentation is a bit confusing—the result in a Straussarian GT is an integrated theoretical structure. The main category in this structure is??? Main concern about what?? Then there are four categories presenting strategies- clarified by the sub-categories…. | Main concept and category: behavior based on faith  Main concern: The challenge of how to do the right thing for the patient  The ethical care process was completely rewritten. It was completely edited. |
| Table 2 make me even more confused—Here the main categories are -Context, Strategies and outcome??? I am not sure what this table 2 is used for. In GT there you have a matrix as a tool in the coding process- otherwise a figure to demonstrate the result. | Was corrected. Several tables and figures have been added to the text to explain the ethical care process. |
| The study aimed to explain the ethical care process of patients with COVID-19. Please, present a result according to Straussarian GT answering your aim. | The results section and, accordingly, the discussion section were completely edited. Corrections are highlighted in blue. |
| The main Category: Striving to create the most ethically valuable care A suggestion is to use the Figure 1 as the starting point and present the findings and explanation from that. | Thank you for your suggestion.  I did the same thing. Fig 1 shows the starting point. Fig 2 shows the entire route. Fig 3 shows the process in more detail. |
| Why are there quotations piled when presenting the categories? This is not the way to present a GT study. | Was corrected. Quotations are included in the description. |
| Please keep focus, the issue under study is about nurses’ ethical concerns in Covid 19 care. There is not much of these perspectives in the result… | It was completely edited. |
| The result section needs to be re-written. This study is focusing on an important and interesting area of research, so please clarify and structure the result. Let the figure guide the writing not necessary state every part- especially when there is only one sentence. | Ok. The results section was completely edited. Added some figures. |
| The discussion starts with another aim, providing ethical care to patients with Covid-19 in order to achieve the desired level of health. | The discussion section was completely edited. Corrections are highlighted in blue. |
| Quite a lot of the text in the discussion is information I would have found in the result. Please move it and clarify the result, then it will be clear to understand the discussion and all the 49 references added in the discussion.(are they all needed?) | The discussion section was completely edited. Corrections are highlighted in blue.  The number of references decreased. |
| There is also an error, in a study conducted by Pazokian in 1401, in a study conducted by Basouli in 1400??? | Was corrected. |
| Limitations are well presented but are lacking methodology issues. However, there are several GT studies conducted about ethical care/approaching. Sorry, but this is not the first one. Qualitative studies are not aiming for comparison, but adding and deepening knowledge. | Limitation section was corrected.  Methodological limitations were also added. Corrections are highlighted in blue.  Yes, there have been several GT studies on moral care, but I wanted to say that grounded theory on ethical care in Covid-19 has not been done so far. |
| Conclusions are presented in appropriate fashion, probably supported by data, but as mentioned previously, the result need to be clarified. I do not agree that the process of providing ethical care to patients with Covid-19 was explained in detail. But this could be corrected. | Was corrected. |
| The article is presented in an intelligible fashion, but the structure could be sharpened, and it is written in standard English, mostly. (the text could be more straight forward) | Was corrected.  The revised text was edited natively by an English company. Its certificate is attached as pdf File. |
| Out of 77 references 18 are 10 years old or more (4 are 9 years old), there are 26 references in the introduction and out of these 7 are 10 years old or more. | Was corrected. All old references were replaced with new references. Only one Corbin and Strauss reference is in 2008, but the rest are from 2017 and above. |
|  |  |
| **Reviewer 2** | **Author's response** |
| I would offer that you have not yet reached the threshold of a grounded theory. The core category "create the most ethically valuable care" does not illustrate the process of how healthcare professionals created ethical care in their work. The core category should tell us what people did to make the process happen. Additionally, the sub categories of jihad, guide, respect, and have compassion and love are descriptive, and do not indicate a process. In grounded theory, the order of the themes matters, showing that a participant moves from one to the next. There are also too many sub themes, indicating there has not yet been enough synthesis and the data needs additional analysis.  I suggest that you could do some additional analysis and refine the theory, then resubmit that that time. You could look at examples from other grounded theories: Jackson, J., Vandall-Walker, V., Vanderspank-Wright, B., Wishart, P., & Moore, S. L. (2018). Burnout and resilience in critical care nurses: A grounded theory of Managing Exposure. Intensive Crit Care Nurs, 48, 28-35. <https://doi.org/10.1016/j.iccn.2018.07.002> | Thank you very much for your suggestion and introducing two excellent articles in this field. I read the articles and understood what you mean. I edited the results section and the discussion section in general. I have tried to express the process of ethical care as a process rather than a description. Corrections are highlighted in blue. |
| I also suggest an edit from a native English speaker prior to resubmission. | Was corrected. The revised text was edited natively by an English company. Its certificate is attached as a pdf File. |
